# Supplementary material for: Resolving the full spectrum of human genome variation using Linked-Reads
Source: Genome Res. 2019 Apr;29(4):635–45. doi: 10.1101/gr.234443.118 (PMC6442396; doi:10.1101/gr.234443.118)
Supplement: Supplemental Material [file supp_gr.234443.118_Supplemental_longranger-2.2.zip › longranger-2.2.tar.bz2!/lr2.2-public/lib/stan/cmdstan/stan/doc/api/html/structstan_1_1gm_1_1add__function__signature.html]

Stan: stan::gm::add\_function\_signature Struct Reference


|  |  |
| --- | --- |
|  | Stan  2.5.0  probability, sampling & optimization |


- Main Page
- Related Pages
- Namespaces
- Classes
- Files

- Class List
- Class Index
- Class Hierarchy
- Class Members

All Classes Namespaces Files Functions Variables Typedefs Enumerations Enumerator Friends Macros Pages

- stan
- gm
- add\_function\_signature

Classes |
Public Member Functions |
Static Public Member Functions |
List of all members

stan::gm::add\_function\_signature Struct Reference

`#include <functions_grammar_def.hpp>`

|  |  |
| --- | --- |
| Classes | |
| struct | result |
|  | |

|  |  |
| --- | --- |
| Public Member Functions | |
| void | operator() (const function\_decl\_def &decl, bool &pass, std::set< std::pair< std::string, function\_signature\_t > > &functions\_declared, std::set< std::pair< std::string, function\_signature\_t > > &functions\_defined, std::ostream &error\_msgs) const |
|  | |

|  |  |
| --- | --- |
| Static Public Member Functions | |
| static bool | fun\_exists (const std::set< std::pair< std::string, function\_signature\_t > > &existing, const std::pair< std::string, function\_signature\_t > &name\_sig) |
|  | |

## Detailed Description

Definition at line 138 of file functions\_grammar\_def.hpp.

## Member Function Documentation

|  |  |  |  |
| --- | --- | --- | --- |
| static bool stan::gm::add\_function\_signature::fun\_exists | ( | const std::set< std::pair< std::string, function\_signature\_t > > & | *existing*, |
|  |  | const std::pair< std::string, function\_signature\_t > & | *name\_sig* |
|  | ) |  |  |

inlinestatic

Definition at line 141 of file functions\_grammar\_def.hpp.

|  |  |  |  |
| --- | --- | --- | --- |
| void stan::gm::add\_function\_signature::operator() | ( | const function\_decl\_def & | *decl*, |
|  |  | bool & | *pass*, |
|  |  | std::set< std::pair< std::string, function\_signature\_t > > & | *functions\_declared*, |
|  |  | std::set< std::pair< std::string, function\_signature\_t > > & | *functions\_defined*, |
|  |  | std::ostream & | *error\_msgs* |
|  | ) |  | const |

inline

Definition at line 153 of file functions\_grammar\_def.hpp.

---

The documentation for this struct was generated from the following file:

- src/stan/gm/grammars/functions\_grammar\_def.hpp


---

[ Stan Home Page ]

*© 2011–2014,
Stan Development Team.*
